# *5-HTTLPR* Expression Outside the Skin: An Experimental Test of the Emotional Reactivity Hypothesis in Children

**DOI:** 10.1371/journal.pone.0141474

**Published:** 2015-11-11

**Authors:** Joyce Weeland, Meike Slagt, Eddie Brummelman, Walter Matthys, Bram Orobio de Castro, Geertjan Overbeek

**Affiliations:** 1 Utrecht Centre for Child and Adolescent Studies, Utrecht University, Utrecht, The Netherlands; 2 Research Institute of Child Development and Education, University of Amsterdam, Utrecht, The Netherlands; 3 Department of Child and Adolescent Studies, Utrecht University, Utrecht, The Netherlands; Sudbury Regional Hospital, CANADA

## Abstract

**Background:**

There is increasing evidence that variation in the promoter region of the serotonin transporter gene SLC6A4 (i.e., the *5-HTTLPR* polymorphism) moderates the impact of environmental stressors on child psychopathology. Emotional reactivity −the intensity of an individual’s response to other’s emotions− has been put forward as a possible mechanism underlying these gene-by-environment interactions (i.e., G×E). Compared to children homozygous for the L-allele (LL-genotypes), children carrying an S-allele (SS/SL-genotypes), specifically when they have been frequently exposed to negative emotions in the family environment, might be more emotionally reactive and therefore more susceptible to affective environmental stressors. However, the association between *5-HTTLPR* and emotional reactivity in children has not yet been empirically tested. Therefore, the goal of this study was to test this association in a large-scale experiment.

**Methods:**

Children (*N* = 521, 52.5% boys, *M*age = 9.72 years) were genotyped and randomly assigned to happy, angry or neutral dynamic facial expressions and vocalizations. Motor and affective emotional reactivity were assessed through children’s self-reported negative and positive affect (*n* = 460) and facial electromyography activity (i.e., *f*EMG: the zygomaticus or “smile” muscle and the corrugator or “frown” muscle, *n* = 403). Parents reported on their negative and positive parenting behaviors.

**Results:**

Children mimicked and experienced the emotion they were exposed to. However, neither motor reactivity nor affective reactivity to these emotions depended on children’s *5-HTTLPR* genotype: SS/SL-genotypes did not manifest any stronger response to emotional stimuli than LL-genotypes. This finding remained the same when taking the broader family environment into account, controlling for kinship, age, gender and genetic ancestry, and when including a tri-allelic factor.

**Conclusions:**

We found no evidence for an association between the *5-HTTLPR* polymorphism and children’s emotional reactivity. This finding is important, in discounting one potential underlying endophenotype of G×E between the *5-HTTLPR* and affective environmental stressors.

## Introduction

When faced with adversity, some children seem particularly vulnerable to develop psychopathology, whereas others seem resilient and show more favorable developmental outcomes. Variability in the promoter region of the serotonin transporter gene SLC6A4 or 5-HTT (i.e., *5-HTTLPR* polymorphism) has been shown to influence the strength of the relationship between environmental adversity and child internalizing and externalizing psychopathology (for an overview see Van IJzendoorn, Belsky, & Bakermans-Kranenburg [1]). However, findings on these gene-by-environment interactions (i.e., G×E) have been mixed. It has been difficult to reliably replicate earlier findings [2–5], and we know little about how these interactions work (i.e., the underlying mechanisms or “how genes get outside the skin”). Emotional reactivity has been put forward as a possible explaining mechanism of the interplay between, specifically, affective environmental risk factors (e.g., angry, harsh parenting, parental martial conflict) and the *5-HTTLPR* [6, 7]. However, the association between *5-HTTLPR* and emotional reactivity has not been empirically tested in children. Moreover, what we know mostly stems from correlational studies, which have their limitations. In a large scale experiment we therefore tested the association between *5-HTTLPR* and children’s motor (i.e., facial electromyography) and affective (i.e., child self-report) emotional reactivity.

### The emotional reactivity hypothesis

Emotional reactivity refers to the form and intensity of an individual’s motor, affective, and sensory response to emotions [8], or in other words, how (strongly) people react to other’s emotions. Emotions are key elements of children’s environment, specifically of parenting, and in turn predictors of their development [9, 10]. Compared to less reactive children, highly reactive children are more likely to respond intensely to an angry or irritated parent [11, 12]: They may more strongly mimic the shown facial expressions (i.e., motor reaction) and experience anger or irritability (i.e., affective reaction). Heightened emotional reactivity (i.e., angry reactivity and irritability) is an important symptom of both Oppositional Defiant Disorder and Child Depression [13], suggesting that it might be an endophenotype underlying both childhood externalizing and internalizing psychopathology. Indeed, individual differences in reactivity to emotional stimuli have been related to both internalizing and externalizing psychopathology [14, 15]. Hence, highly reactive children might be at increased risk for developing psychopathology, when exposed to highly negative emotions.

Individual differences in the processing of emotional stimuli have been associated with variation in availability of the neurotransmitter serotonin, an important modulator of neural circuitry controlling mood [16]. The *5-HTTLPR* polymorphism is a key regulator of this availability [17]. Compared to the high expressing long allele (i.e., L-allele), the low expressing short allele (i.e., S-allele) of this polymorphism has been associated with significantly lower 5-HTT mRNA and protein, lower uptake, and consequently higher and less stable concentrations of serotonin in the synaptic cleft [18, 19] (but see Naylor et al. [20]). Such high and unstable serotonin concentrations might contribute to higher neural reactivity and more intense and prolonged arousal, when people are exposed to emotions [21]. This is illustrated by findings showing that, compared to people homozygous for the L-allele (i.e., LL-genotypes) people carrying at least one S-allele (i.e., SS/SL-genotypes) show heightened amygdala activity in response to emotional stimuli, presumably indicating higher arousal during emotion processing [22, 23]. The *5-HTTLPR* S-allele might thus predispose people to heightened emotional reactivity.

Although we know little about how such neurological arousal is related to the actual experience of emotions and how it eventually “translates” into behavior, previous studies on adults do suggest that S-allele carriers show heightened motor, physiological and affective reactivity after being exposed to an emotional film [24], maternal expressed emotions [12], or unsupportive parenting [25]. One possibility therefore is that the neurological arousal after being exposed to other’s emotions indirectly affects behavior via changes in individual’s emotion expression and mood (or affective state) [26–28]. Indeed, previous research indicates that neural and motor reactivity to emotional stimuli are related, and that in turn these reactivity measures might indicate “emotional contagion” [26–28]. Thus, S-allele carriers might be at increased risk for developing psychopathology when exposed to affective environmental stressors because they show heightened reactivity to these stressors. One pioneering study indeed found the joined effect of the *5-HTTLPR* and family adversity on externalizing symptoms to be mediated by children’s angry reactivity [8]. However, this study examined angry reactivity in particular rather than emotional reactivity in general. As of yet, there has been no previous research on the association between *5-HTTLPR* variability and differences in how strongly children react to other’s emotional expressions with their own emotions.

Underlying endophenotypes such as emotional reactivity are not explained by genetic variation alone. They most likely take the form of entrained bio-psychosocial traits: Partly heritable traits that are shaped by countless interactions with the environment during early development [29]. In turn, these traits can directly contribute to the development of psychopathology, as well as indirectly through the way children react to the environment. In case of emotional reactivity, it seems likely that the *5-HTTLPR* interacts with affective environmental stressors, such as family conflict and highly negative or low-supportive parenting, in eventually predicting individual differences. Children’s observations of marital conflict, for example, have been shown to contribute to an increased sensitivity to anger cues [30]. Repeated exposure to such affective environmental stressors (e.g., low supportive or harsh parenting) possibly alters neurophysiological arousal to such emotional stimuli, in some children more than others [31, 32]. Specifically for SS/SL-genotypes, affective environmental stressors might form a cumulative risk factor for heightened reactivity to negative emotions. Thus, SS/SL-genotypes might be especially emotionally reactive when they have experienced highly negative and low-positive parenting in the past. It has been shown that amygdala and hippocampus activation in rest correlates positively with life stress in SS/SL-genotypes, but negatively in LL-genotypes [33]. Thus, for LL-genotypes (but not SS/SL genotypes) such stressors might form a cumulative risk factor for decreased reactivity to negative emotions (i.e., hypo-arousal, [21]). As a result, LL-genotypes might be especially emotionally *unreactive* when they have experienced highly negative and low-positive parenting in the past.

In sum, emotional reactivity has been put forward as a possible explaining mechanism of the interplay between, specifically, the *5-HTTLPR* and affective environmental risk factors (e.g., angry, harsh parenting, and parental martial conflict) in predicting child psychopathology. By clarifying the underlying mechanisms of G×E we might increase the empirical and clinical value of our knowledge: It may enable us to form specific a priori hypothesis, and specify our research strategies accordingly. Eventually, this might help us to better tailor interventions and increase their effectiveness [34]. However, the association between *5-HTTLPR* and emotional reactivity has not been tested empirically in children. Moreover, existing studies on emotional reactivity have been correlational, which do not permit causal inferences. Here, we report a large scale experiment testing the emotional reactivity hypothesis. In addition to enabling causal inferences, experiments have several important advantages over correlational studies. First, by randomizing participants across experimental conditions, they rule out alternative explanations for G×E [35]. For example, genetic factors and environmental factors, such as parenting, might be correlated because children with a specific genetic make-up evoke specific parenting behaviors or emotions (i.e., gene-environment correlation, or *r*GE). By randomly assigning individuals to conditions, the manipulated variable cannot be related to children’s genetics. Second, experiments prevent the usually highly skewed distributions of environmental risk by manipulating the environmental risk factor (in our case negative emotions). Third, manipulation of the environment creates a standardized, specific and clear environmental stimulus, representing the targeted environmental stressor, and will therefore decrease “noise” in the assessment of G×E and will increase power to detect interactions if present.

### Present study

The present experiment tested whether SS/SL-genotypes were more emotionally reactive than LL-genotypes, specifically SS/SL-genotypes who were frequently exposed to highly aversive or low supportive emotions in the family environment. Standardized video clips of dynamic facial expressions were used to simulate exposure to other’s emotions. Such emotional stimuli have been successfully used in previous studies to evoke an emotional response and enabling measurement of emotional reactivity [36, 37]. Although previous studies on the *5-HTTLPR* have mostly focused on the negative consequences of environmental risk, heightened emotional reactivity may work both ways, as heightened reactivity to both negative (aversive) as well as to positive (supportive) emotions (i.e., for better and for worse [38]). To be able to test for both dual risk (i.e., for worse) as well as differential susceptibility interactions (i.e., for better and for worse), our experiment contained positive as well as negative emotional stimuli, and assessments of positive as well as negative reactivity [38]. To avoid “carry-over” effects of the different emotions, a between-subjects design was selected. Furthermore, because emotional reactivity is a multi-faceted construct it was measured using a multi-method approach. First, by using, a motor reactivity measure consisting of activity measures of the corrugator supercilii muscle -which frowns the brows- and the zygomaticus major muscle -which pulls up the corners of the mouth- (i.e., facial electromyography activity, or *f*EMG). Second, by using an affective reactivity measure consisting of child self-reported happiness, anger, sadness, and fear. Both self-report and *f*EMG have been shown to be valid measures of emotional reactivity in school aged children [39, 40]. However, although part of the same process, such motor and affective reactivity each may play a unique role in the translation of emotional arousal to behavior and are therefore not, or only weakly, related to each other [41, 42].

We expected a main effect of condition (i.e., neutral, happy or anger) on both motor and affective reactivity. Specifically, we expected children exposed to happy emotional expressions and vocalizations to show higher zygomaticus activity (i.e., smile) and report higher positive affect, compared to the anger or neutral condition. Similarly, we expected children exposed to angry facial expressions and vocalization show higher corrugator activity (i.e., frown) and report higher negative affect, compared to the happy or neutral condition. Most importantly, we expected *5-HTTLPR* genotype to moderate the condition-effect, with SS/SL-genotypes showing higher motor and affective reactivity than LL-genotypes. In addition, we expected a three-way interaction of condition-×-*5-HTTLPR* genotype-×-parenting interaction. Here, we expected the condition-effect to be especially strong for SS/SL-genotypes who were exposed to highly negative or low positive parenting in their home situation.

## Methods and Materials

### Participants

Participants were 521 children (52.5% boys) aged 6 to 13 years (*M* = 9.72; *SD* = 1.51) and their parents (61.1% mothers; *M*age = 42.45; *SD* = 4.68), recruited in Science Centre NEMO Amsterdam (The Netherlands), as part of Science Live, an innovative research program that enables scientists to carry out research using NEMO visitors as volunteers. Most children (90.0%) were from Dutch ancestry (i.e., parents were born in The Netherlands) and were living with both parents (84.8%). Over half of parents (63.5%) were higher educated (i.e., completed university or higher vocational training), around a third (30.7%) completed lower vocational training and 5.6% completed primary or high school. Overall, parents reported relatively high positive parenting and low negative parenting (see APQ scores beneath).

### Procedure and instruments

All children received active informed written parental consent. Only children accompanied by their parent(s) or legal guardian(s) were allowed to participate in the study. Children aged 12 and older also signed an active informed consent form. Based on their ID number children were randomly assigned to one of three experimental conditions (i.e., happy, angry or neutral). To avoid “carry-over” effects of emotions, children were exposed to one of the three emotions only. To put children at ease and to let them get familiar with the computer children first answered some simple questions about age and gender. While children were involved in their assessment parents completed a digital questionnaire on parenting behavior in a separate room. We collected a buccal swab for the purpose of genotyping children. This study and the procedures were approved by the Medical Ethical Board of the Utrecht Medical Center (protocol number 12-634/K).

#### Stimulus material

Children watched four standardized clips −each lasting approximately 400 ms− displaying either happy, angry or neutral facial expressions (Amsterdam Dynamic Facial Expression Set [36]). The clips showed two Dutch male and two Dutch female models starting with neutral facial expressions, after which they gradually start to express the target emotion while turning towards the viewer. Viewers in previous studies have indicated feeling more involved with this version compared to the face-forward version [43]. The clips were synchronized with nonlinguistic vocalizations of the target emotion that were intense yet natural: Laughing in the happy condition, growling in the angry condition, or clicking of the tongue in the neutral condition.

#### Motor reactivity: fEMG measure

During exposure to the stimulus material, bipolar EMG recordings were made from children’s left zygomaticus major and left corrugator supercilii muscles (guidelines by Fridlund and Cacioppo [44]), using five 2 mm surface Ag/AgCl electrodes filled with conductive paste. Raw EMG recordings were made with a portable digital recorder for preprocessing and storage of physiological data (Vitaport III, TEMEC Instruments B.V., Kerkrade, The Netherlands). Signals were antialiasing filtered using a 512 Hz lowpass filter and were digitized at 1024 Hz.

#### Affective reactivity: child self-report

Both before and after exposure to the stimulus material (i.e., baseline and response) children answered questions about their affective state (i.e., mood) using a visualized five-point Likert scale (1 = *not at all* to 5 = *totally*, adapted from Reijntjes and colleagues [45]). All 12 items were formulated the same way (e.g., ‘At this moment I feel *angry*’) and together formed four scales capturing the basic emotions of anger, fear, sadness, and happiness. Responses were averaged per emotion. Reliability was satisfactory for all emotions (Cronbach *α*’s between .76 and .85) and did not vary by children’s age. Because of high correlations (*r =* .61 - .71, see Table 1) between the three negative emotions anger, fear, and sadness, for further analyses, we grouped these emotions together to form a negative affect scale (vs. happiness as positive affect scale). Reliability of the negative affect scale was good (Cronbach *α* = .89 at baseline and .91 at response).

**Table 1:** Correlations between Child Genotype, Parenting and Mood.

	1	2	3	4	5	6	7
1. *5-HTTLPR* genotype	-	-.01	.02	-.08	-.03	.01	.00
2. Positive parenting	-	-	-.24**	.03	-.04	.01	.01
3. Negative parenting	-	-	-	-.05	.03	.05	.00
4. Happiness	-	-	-	-	-.27**	-.27**	-.30**
5. Anger	-	-	-	-	-	.63**	.71**
6. Sadness	-	-	-	-	-	-	.61**
7. Fear	-	-	-	-	-	-	-

*Note*.

** *p* <. 01

#### Parenting behavior

The Alabama Parenting Questionnaire (APQ [46]) was used to measure the broader affective family environment (i.e., aversive and supportive parenting). The questionnaire consists of 35 items which make up the scales Involvement (10 items, e.g., ‘You have a friendly talk with your child’), Poor monitoring (10 items, e.g., ‘Your child is out with friends you do not know’), Positive parenting (6 items, e.g., ‘You praise your child if he/she behaves well’), Corporal punishment (3 items, e.g., ‘You slap your child if he/she has done something wrong’), Inconsequent discipline (6 items, e.g., ‘The punishment you give your child depends on your mood’) and Other. Parents filled out how often they use the described practices using a 5-point Likert-scale (1 *= never* to 5 *= always*). It has been shown that negative parenting behavior, such as authoritarian and harsh parenting, is related to negative parental affect and expressed emotions, but that sensitive and encouraging parenting is related to positive parental affect and expressed emotions [47–49]. Therefore, to create scales that measure such parenting behavior we combined the scales involvement and positive parenting to create a positive parenting scale and the scales corporal punishment, poor monitoring and inconsequent discipline to create a negative parenting scale (cf. Prevatt [50]). The APQ ‘other’ scale was not used because of its lack of specificity. Reliability of both scales was good (Cronbach *α* for positive parenting was .81 and for negative parenting was .76). Overall, parents reported relatively high positive parenting (between 2.63 and 4.88; *M* = 3.99; *SD* = .36) and low negative parenting (between 1.06 and 3.00; *M* = 1.77; *SD* = .32).

### Data preparation

#### fEMG measures

Differential *f*EMG signals were filtered offline (high-pas 20 Hz, 48dB/octave) and rectified using Brain Vision Analyzer Software (Brain Products GmbH, Munich). Raw *f*EMG data were segmented into 100 ms epochs. For each clip a baseline (i.e., neutral expression at start) and reaction epoch (i.e., onset of dynamic expression) was selected (cf. Deschamps and colleagues [39]), resulting in four baseline and four reaction epochs per child. Since we applied this very strict *f*EMG baseline measure consisting of a baseline epoch of all four clips, conditions did differ on baseline scores of both muscles (*p*s < .01). These differences can be explained by “carry-over” effects of muscle response to a clip, to the baseline measure of the next clip: Muscle reaction to clip one onto baseline epoch of clip two (etc.). Absolute scores of 3*SD* above the mean were considered extreme outliers and were removed. Due to technical problems and measurement errors (e.g., equipment failure, “noise” from another room, or children wishing to discontinue the *f*EMG procedure), *f*EMG data were missing for 47 children. Children without *f*EMG data did not differ from children with *f*EMG data on age, gender, genetic ancestry, *5-HTTLPR* genotype, or on parent-reported parenting behavior (*p*s > .11).

#### Genotype

Buccal swabs from children were collected in lysisbuffer (100 mM NaCl, 10 mM EDTA, 10 mM Tris pH 8, 0.1 mg/ml proteinase K and 0.5% w/v SDS) until further processing. Genomic DNA was isolated from the samples using the Chemagic buccal swab kit on a Chemagen Module I workstation (Chemagen Biopolymer-Technologie AG, Baesweiler, Germany). The region of interest from the 5-HTT gene was amplified by PCR using the following primers: a FAM-labelled primer 5’-TCCTCCGCTTTGGCGCCTCTTCC-3’, and a reverse primer 5’-TGGGGGTTGCAGGGGAGATCCTG-3’. Typical PCR reactions contained between 10 and 100 ng genomic DNA templates, 10 pmol of forward and reverse primer. PCR was carried out in the presence of 5% DMSO, 5x buffer supplied with the enzyme and with 1.25U of LongAmp *Taq* DNA Polymerase (NEB) in a total volume of 30 μl using the following cycling conditions: initial denaturation step of 10 min at 95^°^C, followed by 26 cycles of 30 sec 95^°^C, 30 sec 69^°^C, 60 sec 65^°^C and a final extension step of 10 min 65^°^C. After PCR 10 μl of the sample is subjected to restriction digestion with the enzyme HpaII in a total volume of 20 μl. Restriction is incubated for 2 hours at 37°C and inactivated by incubating for 20 min. at 80°C. One microliter of PCR product before and after restriction was mixed separately with 0.3 μl LIZ-500 size standard (Applied Biosystems) and 11.7 μl formamide (Applied Biosystems) and run on an AB 3730 genetic analyser set up for fragment analyses with 50 cm capillaries. Results were analysed using GeneMarker software (Softgenetics).

Saliva of 513 children (36.8% LL-genotypes; 47.4% SL-genotypes; 15.8% SS-genotypes) was successfully genotyped. Hardy-Weinberg equilibrium (HWE) proportions were estimated [51], and no deviations from these proportions were found (*χ* ^*2*^ = 0.04; df = 2; *p* = .89). For analyses, assuming the S-allele is dominant [18], the *5-HTTLPR* was dummy coded (i.e., SS and SL vs. LL). Possible associations between genotype and mood and possible Gene-Environment correlations (i.e., *r*GE) were checked. No significant correlations between genotype and parenting or child mood (i.e., pre-test scores) were found (see Table 1).

## Results

### Randomization check

Chi square and ANOVA tests showed no differences between conditions on children’s age, gender, *5-HTTLPR* genotype, genetic ancestry, and child reported emotions at baseline or on parent-reported parenting (*p*s > .21), indicating successful randomization.

### Overall effects of condition stimuli

We examined the effect of experimental condition using MANOVA’s with condition as predictor, controlling for baseline *f*EMG activity and self-reported affect scores. To control for skewed distributions we used a bootstrapping procedure. As reported by previous studies [41, 42], motor and affective reactivity were not systematically related (small significant correlations were only found between corrugator and anger response (*r* = .11, *p* < .05), between zygomaticus and fear response (*r =* .11, *p* < .05), and between zygomaticus and anger response (*r =* .14, *p* < .01)). Therefore, separate analyses were conducted for motor and affective emotional reactivity. Analyses showed that the condition stimuli has a small but significant effect on both motor (*N* = 449; Wilks’Lambda = .90, *F*(4, 886) = 12.55, *p* < .001, partial η² = .05) and affective reactivity (*N* = 518; Wilks’Lambda = .93 *F*(4, 1024) = 9.43, *p* < .001 partial η² = .04) (see Table 2 for mean differences). As expected, children in the happy condition showed highest zygomaticus activity response (i.e., strongest smile) and highest response score for positive affect (i.e., happiness), but lowest for negative affect (i.e., anger, sadness and fear) of all conditions. Children in the angry condition showed highest corrugator activity response (i.e., strongest frown) and lowest response score for positive affect, but highest for negative affect of all conditions. Overall, these findings showed children’s tendency to mimic and experience the emotion they were exposed to, indicating successful manipulation of emotions. Consistent with previous research [52], the manipulation evoked stronger motor (but not affective) reactivity in girls (Partial *η²* = .11), compared to boys (Partial *η²* = .04) (see under auxiliary analyses and Tables A and B in S1 Table).

**Table 2:** Descriptive Statistics of Motor Reactivity (Zygomaticus and Corrugator Muscle activity) and Affective Reactivity (Self-reported positive and negative affect).

	Happy (*n* = 170)						Angry (n = 172)						Neutral (n = 179)					
	Total		SS/SL		LL		Total		SS/SL		LL		Total		SS/SL		LL	
Variable	*M*	*SD*	*M*	*SD*	*M*	*SD*	*M*	*SD*	*M*	*SD*	*M*	*SD*	*M*	*SD*	*M*	*SD*	*M*	*SD*
Zygomaticus	12.13 ^a^ ^b^	7.4	10.61	6.31	14	8.25	10.40 ^c^	6.4	10.73	6.92	9.84	7.1	7.06^c^	6.56	7.7	7.18	5.8	5.2
Corrugator	6.11 ^a^ ^b^	3.2	6.6	3.71	5.5	2.16	9.42 ^c^	5.2	9.84	5.17	8.4	5.2	7.51^c^	6.31	7.4	6.1	7.7	6.7
Positive affect	4.27^b^	0.9	4.38	0.74	4.1	0.98	4.01 ^a^ ^c^	0.9	3.96	0.99	4.1	0.8	4.21^b^	0.82	4.2	0.87	4.2	0.7
Negative affect	1.26 ^b^	0.6	1.23	0.52	1.3	0.67	1.46 ^a^ ^c^	0.7	1.5	0.76	1.38	0.6	1.32^b^	0.6	1.4	0.68	1.3	0.5

^a^ Significantly different from neutral

^b^ Significantly different from anger

^c^ Significantly different from happy

### Moderation effects of genotype and parenting

We examined the effect of genotype and parenting using MANOVA’s, again controlling for baseline and skewed distributions. To avoid multicollinearity in the interaction analyses, mean-centered scores were computed for positive and negative parenting scales. See the results in Tables 3 and 4. Crucially, the two-way condition×genotype interaction was not significant, indicating there is no direct relation between the *5-HTTLPR* and emotional reactivity. Thus, contrary to our hypotheses, the strength of children’s motor and affective reactions to the emotion they were exposed to did not depend on genotype. The three-way condition×genotype×parenting interactions were also not significant, indicating that *5-HTTLPR* did not moderate emotional reactivity, regardless of children’s broader affective family environment (i.e., positive and negative parenting).

**Table 3:** Multivariate Results Motor Reactivity to Condition Stimuli.

	*Wilk’s Lambda*	*F*	*df*	*dferror*	*p*	Partial η²
*All children with complete data (n = 403)*						
Condition	.95	2.51	8.00	760.00	.01	.03
*5-HTTLPR*	1.00	.66	4.00	379.00	.62	.01
Negative parenting	1.00	.36	4.00	383.00	.62	.00
Positive parenting	.98	3.66	2.00	383.00	.03	.02
Condition×*5-HTTLPR*	.99	1.17	4.00	764.00	.33	.01
Condition×negative parenting	1.00	.20	4.00	766.00	.94	.00
Condition×positive parenting	.99	1.01	4.00	766.00	.81	.00
Condition×*5-HTTLPR×*negative parenting	.98	2.08	4.00	766.00	.08	.01
Condition×*5-HTTLPR*×positive parenting	1.00	.41	4.00	766.00	.81	.00

**Table 4:** Multivariate Results Affective Reactivity to Condition Stimuli.

	*Wilk’s Lambda*	*F*	*df*	*dferror*	*p*	Partial η²
*All children with complete data (n = 460)*						
Condition	.95	5.86	4.00	878.00	.00	.03
*5-HTTLPR*	1.00	.51	2.00	439.00	.60	.00
Negative parenting	1.00	.04	2.00	439.00	.96	.00
Positive parenting	.97	6.33	2.00	439.00	.00	.03
Condition×*5-HTTLPR*	1.00	.44	4.00	878.00	.78	.00
Condition×negative parenting	.99	1.44	4.00	878.00	.22	.01
Condition×positive parenting	.99	.88	4.00	878.00	.48	.00
Condition×*5-HTTLPR*×negative parenting	.99	.77	4.00	878.00	.55	.00
Condition×*5-HTTLPR*×positive parenting	.99	1.01	4.00	878.00	.40	.01

#### Auxiliary analyses

To address factors possibly confounding the results, we performed four auxiliary analyses. First, to control for possible effects of family-relations between participants (i.e., control for kinship), we randomly included one parent-child dyad per family. Second, to control for possible systematic differences in allele frequencies within subgroups from different genetic ancestry (i.e., population stratification [53]) we excluded children of whom one or both parents were not born in Europe. Third, assessing genetic variation using a single polymorphism has been criticized. Multiple polymorphisms within a gene might alter gene expression. Specifically, the *5-HTTLR* L-allele has an embedded single nucleotide substitution (SNP rs25531) (A>G), which seems to inhibit transcription, creating an allele functionally equivalent to the S-allele [54]. To control for possible effects of this SNP on *5-HTTLPR* expression we repeated our analyses using a tri-allelic genetic factor (cf. Braithwaite and colleagues [3]): low expression: SS, SL_G_, L_G_L_G_ (16.40%); medium expression: SL_A_, L_G_L_A_ (54.40%); and high expression: L_A_L_A_ (29.20%)_._ Weinberg equilibrium (HWE) proportions were estimated (87.9% AA genotype) [51], and no deviations from these proportions were found (*χ* ^*2*^ *=* .50; df = 2; *p* = .78). Fourth, in adults opposing effects have been found regarding the *5-HTTLPR* for males and females in the development of internalizing psychopathology. For females, childhood stressors predicted higher depression in S-allele carriers, whereas for males this was in LL-genotypes [55]. It might therefore be that different mechanisms underlie G×E including the *5-HTTLPR* in males and females. There are indeed indications that there are gender differences in serotonergic functioning, specifically in central nervous system (CNS) serotonin turnover [56–58]. Also, it might be that there are sensitive periods (i.e., effects of G×E might be stronger in some developmental periods than in others), or even critical periods (i.e., effects of G×E might be only present in certain developmental periods) for specific G×E [59]. To control for possible confounding effects of child gender and age we repeated the analyses including condition×genotype×age and condition×genotype×gender interactions.

In all abovementioned analyses, the condition×genotype and the condition×genotype×parenting interactions remained non-significant, regardless of kinship, genetic ancestry, possible tri-allelic effects, age and gender (Tables A and B in S1 Table). Thus, these analyses too showed that the strength of children’s motor and affective reactivity to the emotion they were exposed to did not depend on their genotype–regardless of whether children’s broader affective family environment (i.e., parenting) was taken into account.

## Discussion

Emotional reactivity has been put forward as a possible mechanism underlying interactions between the *5-HTTLPR* polymorphism and affective environmental risk (e.g., angry, harsh parenting) in predicting child psychopathology. If emotional reactivity indeed functions as an explanatory mechanism, it should be directly associated with the *5-HTTLPR* polymorphism, specifically in interaction with the broader environment children grow up in. Knowledge on such mechanisms underlying G×E is important to increase the empirical and clinical value of the discoveries made so far [60, 61]. In a large-scale experiment we tested whether children’s motor and affective reactivity to dynamic facial expressions and vocalizations was dependent on their *5-HTTLPR* genotype. Results showed that this is not the case: Although children reacted to the happy, angry and neutral stimuli in an emotionally congruent manner, this emotional reactivity was not associated with the *5-HTTLPR* polymorphism, regardless of children’s broader affective environment (i.e., positive and negative parenting). This finding did not change controlling for kinship, genetic ancestry, age, gender or including a tri-allelic genetic factor. Together, these findings challenge the hypothesis that emotional reactivity underlies interactions between the *5-HTTLPR* polymorphism and affective environmental stressors in predicting child psychopathology.

How do our findings relate to previous research? Although previous studies found S-allele carriers to show heightened neural response to emotional stimuli [23, 24], we did not find these genetic differences to translate to heightened motor or affective reactions in children. Consequently, our findings do not confirm that emotional reactivity, as has been theorized, is indeed an underlying mechanism of previous found G×E including the *5-HTTLPR* polymorphism [6, 7]. This contradicts earlier findings, showing that children’s angry reactivity mediated the interaction between *5-HTTLPR* and maternal unresponsiveness [7]. One possibility is that emotional reactivity −as we measured it− is simply not a mechanism underlying G×E including the *5-HTTLPR*. The *5-HTTLPR* has been related to other affective, cognitive and behavioural endophenotypes, besides emotional reactivity, as well [6, 62]. G×E including the 5*-HTTLPR* might therefore result, not from differences in emotional reactivity, but rather from differences in the ability to *regulate* emotions evoked by others (e.g., how children reframe or suppress these emotions). Indeed, a recent study found that emotion regulation (i.e., using cognitive reappraisal) served as a buffer in the association between *5-HTTLPR*, environmental stressors, and psychopathology [63]. Because the distinction between emotional reactivity, regulation and processing (e.g., cognitive processes) is a difficult one [64, 65], it might be conducive for future research efforts to take into account different emotional processes that are possibly involved in individual differences in psychopathology.

It is important to interpret our present results in light of specific limitations. First, being hosted by a Research Centre −an educative attraction− caused an oversampling of relatively highly educated and well-adjusted families. The range of reported parenting behavior was therefore limited to scores reflecting (sub)optimal behavior. Our results might not be generalizable to the general population. Future research should therefore test these hypotheses in more heterogeneous samples and also in at risk samples. Additionally, to avoid “carry-over” effects from one emotion to another a between subject design was selected. This means our study does not actually measure whether the same children genetically differ in emotional reactivity to both positive *and* negative emotions (i.e., for better and for worse), but only whether children differ in reactivity to either negative *or* positive emotions. Furthermore, the emotional reactivity hypothesis is based on differences in serotonin availability, which is influenced by multiple genes but which was measured in this study by *5-HTTLPR* variability alone. Including more genetic factors possibly influencing serotonin availability could be an important next step. For example, a serotonergic genetic pathway (i.e., also including genes coding for serotonin synthesis and reuptake, such as *TPH1*, *HTR1A*, and *HTR2C*) could be used as moderator.

Nevertheless, our study design has some important strengths. First of all, our study is part of the growing body of experimental research on G×E (for an overview see Bakermans-Kranenburg and Van IJzendoorn [66]) providing more robust measures of G×E compared to correlational designs (e.g., ruling out alternative explanations for such an interaction). Second, our large scale genetically informed experiment shows it is possible to manipulate emotions in school-aged children and to measure their emotional reactions through both self-report and *f*EMG. We used a multi-method multi-informant approach, using both observed motor and self-reported affective reactivity measures. Replicating previous research [26, 67], we found stronger motor (but not affective) reactivity in girls than boys, which might indicate gender differences in emotion processing. Third, our study assessed children’s reactivity to standardized and precise measures of both negative (i.e., anger) and positive (i.e., happiness) affective environmental factors (i.e., for better and for worse). We tested whether children genetically differ in their general emotional reactivity by exposing them to standardized emotional facial expressions, which were directed toward the child, in a controlled laboratory setting rather than in “real life” situations. However, this approach might explain why our findings differ from those of Davies and Cicchetti [7] (who exposed children to a simulated angry phone conversation between the mother and her intimate partner) and Gyurak and colleagues [24] (who exposed adults to a clip about the Darfur crisis). These previous studies did not include standardized emotion expressions addressing the child.

Studies on G×E in psychopathology form a fast growing field of research, yielding exciting results on the interplay between genes and environment. However, we have little understanding of how such interactions work. In order for these interactions to have empirical and clinical implications, we need to understand the underlying psycho-biological mechanisms. Our findings showed that one important hypothesis on why children might be more susceptible to environmental stressors did not hold true: Contrary to the emotional reactivity hypothesis, children carrying a SS/SL-genotypes of the *5-HTTLPR* were not more emotionally reactive than LL-genotypes. Our findings might indicate that previously found differences between SS/SL- and LL-genotypes in emotion processing do not translate into differences in motor and affective reactivity. Future research should explore whether such processes rather translate into other psycho-biological processes such as emotion regulation or processing. Unravelling mechanisms underlying G×E demands that we report both findings that confirm and findings that falsify our theories or assumptions about specific G×E. Only such transparency can help us gain insight into the validity and specificity of these mechanisms (see also Heininga et al., 2015 on ambivalent G×E findings including the *5-HTTLPR* [5]). However, we must keep in mind that the absence of evidence is not necessarily the evidence of absence. In this regard, it is pivotal to acknowledge that research on the interplay between genetics and the environment is making important (technological) progress. Future studies using epigenetics, haplotypes and the whole genome (i.e., GWAS) might yield more solid results [67–69]. Such strategies might also prove to be powerful designs to further explore the mechanisms of G×E.

To summarize, we conclude that how strongly children react to other’s emotions, with their facial expression and own affect, is neither directly explained by variation in the *5-HTTLPR*, nor by a combination of *5-HTTLPR* variability and previous interactions with the environment. These findings challenge the emotional reactivity hypothesis.

## Supporting Information

S1 Dataset Dataset *5-HTTLR* and emotional reactivity. (CSV)

S1 File Codebook dataset. (PDF)

S1 Table Auxiliary analyses Table A and B. (DOC)
