# Supplementary material for: 5-HTTLPR Expression Outside the Skin: An Experimental Test of the Emotional Reactivity Hypothesis in Children
Source: PLoS One. 2015 Nov 11;10(11):e0141474. doi: 10.1371/journal.pone.0141474 (PMC4641607; doi:10.1371/journal.pone.0141474)
Supplement: S1 File — (PDF) [file pone.0141474.s002.pdf]

Variable Information

Variable	Position	Label	Measurement Level	Role	Column Width
ID	1	ID number	Scale	Input	8
Condition	2	Condition	Nominal	Input	19
Brusje	3	Filter kinship	Nominal	Input	8
Relation	4	Relation parent to child	Nominal	Input	10
Sex_c	5	Gender child	Nominal	Input	8
Date_birth_C	6	Date of birth child	Scale	Input	8
Date_birth_P	7	Date of birth parent	Scale	Input	8
age_c	8	age child	Scale	Input	10
age_p	9	age parent	Scale	Input	10
Family	10	Living situation family	Nominal	Input	8
GebOuder1	11	Place of birth parent 1	Nominal	Input	11
GebOuder2	12	Place of birth parent 2	Nominal	Input	11
GebOuders	13	Filter genetic ancestry	Nominal	Input	11
Education	14	Parental education	Nominal	Input	8
Employment	15	Parental employment	Nominal	Input	8
SNPAllel1	16	SNP allele 1	Nominal	Input	12
SNPAllel2	17	SNP allele 2	Nominal	Input	14
@5HTTallel1	18	VNTR allele 1	Nominal	Input	12
@5HTTallel2	19	VNTR allele 2	Nominal	Input	12
@TRIALLELIC	20	Tri-alleltic factor	Nominal	Input	13
@5HTTgen	21	5-HTTLPR VNTR genotype	Nominal	Input	10
@SNP	22	5-HTTLPR SNP genotype	Nominal	Input	10
preBang	23	self-reported pre fear	Nominal	Input	8
preBedroefd	24	self-reported pre sad	Nominal	Input	8
preBezorgd	25	self-reported pre fear	Nominal	Input	8

Variable Information

Variable	Alignment	Print Format	Write Format	Missing Values
ID	Right	F40	F40	999
Condition	Right	F8	F8	999
Brusje	Right	F8.2	F8.2	999.00
Relation	Right	F8.2	F8.2	999.00
Sex_c	Right	F8	F8	999
Date_birth_C	Left	DATE20	DATE20	04-Oct-1582, 04-Oct-1582
Date_birth_P	Left	DATE20	DATE20	04-Oct-1582, 04-Oct-1582
age_c	Right	F8.2	F8.2	999.00
age_p	Right	F8.2	F8.2	999.00
Family	Left	F12	F12	999, 888
GebOuder1	Right	F8.2	F8.2	999.00
GebOuder2	Right	F8.2	F8.2	999.00
GebOuders	Right	F8.2	F8.2	999.00
Education	Left	F12	F12	999, 888
Employment	Left	F12	F12	999, 888
SNPAllel1	Right	F12	F12	999
SNPAllel2	Right	F1	F1	999
@5HTTallel1	Right	F12	F12	999
@5HTTallel2	Right	F1	F1	999
@TRIALLELIC	Right	F8.2	F8.2	999.00
@5HTTgen	Right	F8	F8	999
@SNP	Right	F8.2	F8.2	999.00
preBang	Right	F8.2	F8.2	999.00
preBedroefd	Right	F8.2	F8.2	999.00
preBezorgd	Right	F8.2	F8.2	999.00

Variable Information

Variable	Position	Label	Measurement Level	Role	Column Width
preBlij	26	self-reported pre happy	Nominal	Input	8
preBoos	27	self-reported pre anrgy	Nominal	Input	8
preDroevig	28	self-reported pre sad	Nominal	Input	8
preGelukkig	29	self-reported pre happy	Nominal	Input	8
preKwaad	30	self-reported pre angry	Nominal	Input	8
preOngerust	31	self-reported pre fear	Nominal	Input	8
preSlecht	32	self-reported pre angry	Nominal	Input	8
preVerdrietig	33	self-reported pre sad	Nominal	Input	8
preVrolijk	34	self-reported pre happy	Scale	Input	8
postBang	35	self-reported post fear	Nominal	Input	8
postBedroefd	36	self-reported post sad	Nominal	Input	8
postBezorgd	37	self-reported post fear	Nominal	Input	8
postBlij	38	self-reported post happy	Nominal	Input	8
postBoos	39	self-reported post angry	Nominal	Input	8
postDroevig	40	self-reported post sad	Nominal	Input	8
postGelukkig	41	self-reported post happy	Nominal	Input	8
postKwaad	42	self-reported post angry	Nominal	Input	8
postOngerust	43	self-reported post fear	Nominal	Input	8
postSlecht	44	self-reported post angry	Nominal	Input	8
postVerdrietig	45	self-reported post sad	Nominal	Input	8
postVrolijk	46	self-reported post happy	Nominal	Input	8
MPreSad	47	Mean pre sad	Scale	Input	10
MPreAngry	48	Mean pre angry	Scale	Input	11
MPreHappy	49	Mean pre happy	Scale	Input	11

Variable Information

Variable	Alignment	Print Format	Write Format	Missing Values
preBlij	Right	F8.2	F8.2	999.00
preBoos	Right	F8.2	F8.2	999.00
preDroevig	Right	F8.2	F8.2	999.00
preGelukkig	Right	F8.2	F8.2	999.00
preKwaad	Right	F8.2	F8.2	999.00
preOngerust	Right	F8.2	F8.2	999.00
preSlecht	Right	F8.2	F8.2	999.00
preVerdrietig	Right	F8.2	F8.2	999.00
preVrolijk	Right	F8.2	F8.2	999.00
postBang	Right	F8.2	F8.2	999.00
postBedroefd	Right	F8.2	F8.2	999.00
postBezorgd	Right	F8.2	F8.2	999.00
postBlij	Right	F8.2	F8.2	999.00
postBoos	Right	F8.2	F8.2	999.00
postDroevig	Right	F8.2	F8.2	999.00
postGelukkig	Right	F8.2	F8.2	999.00
postKwaad	Right	F8.2	F8.2	999.00
postOngerust	Right	F8.2	F8.2	999.00
postSlecht	Right	F8.2	F8.2	999.00
postVerdrietig	Right	F8.2	F8.2	999.00
postVrolijk	Right	F8.2	F8.2	999.00
MPreSad	Right	F8.2	F8.2	999.00
MPreAngry	Right	F8.2	F8.2	999.00
MPreHappy	Right	F8.2	F8.2	999.00

Variable Information

Variable	Position	Label	Measurement Level	Role	Column Width
MPreFear	50	Mean pre fear	Scale	Input	10
MPostSad	51	Mean post sad	Scale	Input	10
MPostAngry	52	Mean post angry	Scale	Input	12
MPostHappy	53	Mean post happy	Scale	Input	12
MPostFear	54	Mean post fear	Scale	Input	11
EMG	55	EMG succesfull yes/no	Nominal	Input	8
MCorBase	56	Mean Baseline Corrugator	Scale	Input	8
MZygoBase	57	Mean Baseline Zygomaticus	Scale	Input	8
MCorResp	58	Mean Respons Corrugator	Scale	Input	8
MZygoResp	59	Mean Respons Zygomaticus	Scale	Input	8
APQ1_A	60	Alabama Parenting Questionnaire : U heeft een gezellig gesprek met uw kind	Nominal	Input	8
APQ1_B	61	U laat het uw kind weten als hij/zij zijn/h	Nominal	Input	8
APQ1_C	62	U dreigt uw kind te straffen, maar straft h	Nominal	Input	8
APQ1_D	63	U helpt als vrijwilliger mee met speciale a	Nominal	Input	8

Variable Information

Variable	Alignment	Print Format	Write Format	Missing Values
MPreFear	Right	F8.2	F8.2	999.00
MPostSad	Right	F8.2	F8.2	999.00
MPostAngry	Right	F8.2	F8.2	999.00
MPostHappy	Right	F8.2	F8.2	999.00
MPostFear	Right	F8.2	F8.2	999.00
EMG	Right	F8.2	F8.2	999.00
MCorBase	Right	F8.3	F8.3	999.000, 666.000
MZygoBase	Right	F8.3	F8.3	999.000, 666.000
MCorResp	Right	F8.3	F8.3	999.000, 666.000
MZygoResp	Right	F8.3	F8.3	999.000, 666.000
APQ1_A	Left	F12	F12	999, 888
APQ1_B	Left	F12	F12	999, 888
APQ1_C	Left	F12	F12	999, 888
APQ1_D	Left	F12	F12	999, 888

Variable Information

Variable	Position	Label	Measurement Level	Role	Column Width
APQ1_E	64	U beloont uw kind of geeft hem/haar iets ex	Nominal	Input	8
APQ1_F	65	Het lukt uw kind niet om een briefje achter	Nominal	Input	8
APQ1_G	66	U speelt spelletjes of doet andere leuke di	Nominal	Input	8
APQ1_H	67	Uw kind haalt u over om hem/haar niet te st	Nominal	Input	8
APQ1_I	68	U vraagt uw kind over zijn/haar dag op scho	Nominal	Input	8
APQ1_J	69	Uw kind blijft ' s avonds later buiten dan d	Nominal	Input	8
APQ1_K	70	U helpt uw kind met zijn/haar huiswerk	Nominal	Input	8
APQ1_L	71	U heeft het gevoel dat het meer problemen o	Nominal	Input	8
APQ1_M	72	U geeft uw kind een complimentje als hij/zi	Nominal	Input	8
APQ1_N	73	U vraagt uw kind wat zijn/haar plannen zijn	Nominal	Input	8
APQ1_O	74	U brengt uw kind met de auto, fiets of het	Nominal	Input	8

Variable Information

Variable	Alignment	Print Format	Write Format	Missing Values
APQ1_E	Left	F12	F12	999, 888
APQ1_F	Left	F12	F12	999, 888
APQ1_G	Left	F12	F12	999, 888
APQ1_H	Left	F12	F12	999, 888
APQ1_I	Left	F12	F12	999, 888
APQ1_J	Left	F12	F12	999, 888
APQ1_K	Left	F12	F12	999, 888
APQ1_L	Left	F12	F12	999, 888
APQ1_M	Left	F12	F12	999, 888
APQ1_N	Left	F12	F12	999, 888
APQ1_O	Left	F12	F12	999, 888

Variable Information

Variable	Position	Label	Measurement Level	Role	Column Width
APQ2_A	75	U prijst uw kind als hij/zij zich goed gedraagt	Nominal	Input	8
APQ2_B	76	Uw kind is weg met vrienden die u niet kent	Nominal	Input	8
APQ2_C	77	U knuffelt of kust uw kind als hij/zij iets goed heeft gedaan	Nominal	Input	8
APQ2_D	78	Uw kind gaat weg zonder een afgesproken tijd om thuis te zijn	Nominal	Input	8
APQ2_E	79	U praat met uw kind over zijn/ haar vrienden	Nominal	Input	8
APQ2_F	80	Uw kind is na het donker buiten zonder een volwassene bij zich	Nominal	Input	8
APQ2_G	81	U stopt eerder met een straf van uw kind (zoals het eerder opheffen van	Nominal	Input	8
APQ2_H	82	Uw kind helpt mee om plannen te maken voor gezinsactivitei ten	Nominal	Input	8

Variable Information

Variable	Alignment	Print Format	Write Format	Missing Values
APQ2_A	Left	F12	F12	999, 888
APQ2_B	Left	F12	F12	999, 888
APQ2_C	Left	F12	F12	999, 888
APQ2_D	Left	F12	F12	999, 888
APQ2_E	Left	F12	F12	999, 888
APQ2_F	Left	F12	F12	999, 888
APQ2_G	Left	F12	F12	999, 888
APQ2_H	Left	F12	F12	999, 888

Variable Information

Variable	Position	Label	Measurement Level	Role	Column Width
APQ2_I	83	U krijgt het zo druk dat u vergeet waar uw kind is of wat hij/ zij aan	Nominal	Input	8
APQ2_J	84	Uw kind wordt niet gestraft als hij/ zij iets fout heeft gedaan	Nominal	Input	8
APQ2_K	85	U gaat naar ouderavonden of andere bijeenkomsten op de school van uw ki	Nominal	Input	8
APQ2_L	86	U vertelt uw kind dat u het fijn vindt als hij/ zij u meehelpt rondom h	Nominal	Input	8
APQ2_M	87	U controleert niet of uw kind thuis komt op de tijd waarop hij/ zij ver	Nominal	Input	8
APQ2_N	88	U vertelt uw kind niet waar u heengaat	Nominal	Input	8
APQ2_O	89	Uw kind komt meer dan een uur later van school dan u hem/haar verwacht	Nominal	Input	8
APQ3_A	90	De straf die u uw kind geeft hangt af van uw stemming	Nominal	Input	8

Variable Information

Variable	Alignment	Print Format	Write Format	Missing Values
APQ2_I	Left	F12	F12	999, 888
APQ2_J	Left	F12	F12	999, 888
APQ2_K	Left	F12	F12	999, 888
APQ2_L	Left	F12	F12	999, 888
APQ2_M	Left	F12	F12	999, 888
APQ2_N	Left	F12	F12	999, 888
APQ2_O	Left	F12	F12	999, 888
APQ3_A	Left	F12	F12	999, 888

Variable Information

Variable	Position	Label	Measurement Level	Role	Column Width
APQ3_B	91	Uw kind is thuis zonder volwassen toezicht	Nominal	Input	8
APQ3_C	92	U geeft uw kind een tik voor haar billen wanneer hij/zij iets fout heef	Nominal	Input	8
APQ3_D	93	U negeert uw kind, wanneer hij/ zij zich aan het misdragen is	Nominal	Input	8
APQ3_E	94	U geeft uw kind een klap met uw hand, wanneer hij/ zij iets fout heeft	Nominal	Input	8
APQ3_F	95	Als straf neemt u privileges of geld van uw kind weg	Nominal	Input	8
APQ3_G	96	Als straf stuurt u uw kind naar zijn/ haar kamer	Nominal	Input	8
APQ3_H	97	U slaat uw kind met een riem, (rij) zweep of ander voorwerp als hij/ zij	Nominal	Input	8

Variable Information

Variable	Alignment	Print Format	Write Format	Missing Values
APQ3_B	Left	F12	F12	999, 888
APQ3_C	Left	F12	F12	999, 888
APQ3_D	Left	F12	F12	999, 888
APQ3_E	Left	F12	F12	999, 888
APQ3_F	Left	F12	F12	999, 888
APQ3_G	Left	F12	F12	999, 888
APQ3_H	Left	F12	F12	999, 888

Variable Information

Variable	Position	Label	Measurement Level	Role	Column Width
APQ3_I	98	U gilt of schreeuwt tegen uw kind als hij/ zij iets verkeerd heeft geda	Nominal	Input	8
APQ3_J	99	Als uw kind zich misdraagt, legt u hem/ haar rustig uit waarom zijn/ ha	Nominal	Input	8
APQ3_K	100	Als straf zet u uw kind apart (op de gang of in een hoek van de kamer)	Nominal	Input	8
APQ3_L	101	Als straf geeft u uw kind extra karweitjes	Nominal	Input	8
M_APQ_PP	102	Mean positive parenting	Scale	Input	10
M_APQ_CP	103	Mean corporal punishment	Scale	Input	10
M_APQ_ID	104	Mean inconsequent discipline	Scale	Input	10
M_APQ_I	105	Mean involvement	Scale	Input	10
M_APQ_PM	106	Mean parental monitoring	Scale	Input	10
M_APQ_O	107	Mean other	Scale	Input	10
M_PP	108	SCALE positive parenting	Scale	Input	10
M_NP	109	SCALE negative parenting	Scale	Input	10
M_NP_CEN	110	Centered negative parenting	Scale	Input	10

Variable Information

Variable	Alignment	Print Format	Write Format	Missing Values
APQ3_I	Left	F12	F12	999, 888
APQ3_J	Left	F12	F12	999, 888
APQ3_K	Left	F12	F12	999, 888
APQ3_L	Left	F12	F12	999, 888
M_APQ_PP	Right	F8.2	F8.2	999.00, 888.00
M_APQ_CP	Right	F8.2	F8.2	999.00, 888.00
M_APQ_ID	Right	F8.2	F8.2	999.00, 888.00
M_APQ_I	Right	F8.2	F8.2	999.00
M_APQ_PM	Right	F8.2	F8.2	999.00
M_APQ_O	Right	F8.2	F8.2	999.00, 888.00
M_PP	Right	F8.2	F8.2	999.00
M_NP	Right	F8.2	F8.2	999.00
M_NP_CEN	Right	F8.2	F8.2	999.00

Variable Information

Variable	Position	Label	Measurement Level	Role	Column Width
M_PP_CEN	111	Centered positive parenting	Scale	Input	10
MPreNA	112	Prescore negative affect	Scale	Input	10
MPostNA	113	Postscore negative affect	Scale	Input	10

Variable Information

Variable	Alignment	Print Format	Write Format	Missing Values
M_PP_CEN	Right	F8.2	F8.2	999.00
MPreNA	Right	F8.2	F8.2	999.00
MPostNA	Right	F8.2	F8.2	999.00

Variables in the working file
