# Supplementary material for: 5-HTTLPR Expression Outside the Skin: An Experimental Test of the Emotional Reactivity Hypothesis in Children
Source: PLoS One. 2015 Nov 11;10(11):e0141474. doi: 10.1371/journal.pone.0141474 (PMC4641607; doi:10.1371/journal.pone.0141474)
Supplement: S1 Table — (DOC) [file pone.0141474.s003.doc]

**Supportive Information 1. Auxiliary analyses.**

Table A. *Multivariate results auxiliary analyses motor reactivity to condition stimuli.*

|  | *Wilk’s Lambda* | | *F* | *df* | *dferror* | | *p* | Partial η² |  | |  |
| --- | --- | --- | --- | --- | --- | --- | --- | --- | --- | --- | --- |
| *Including one parent-child dyad per family (n = 360)* | | | | | | | |  |  | |  |
| Condition | | .90 | 8.85 | 4.00 | 680.00 | .00 | | .05 |  | |  |
| *5-HTTLPR* | | 1.00 | .74 | 2.00 | 340.00 | .48 | | .00 |  | |  |
| Negative parenting | | 1.00 | 1.04 | 2.00 | 340.00 | .35 | | .01 |  | |  |
| Positive parenting | | .99 | 1.98 | 2.00 | 340.00 | .14 | | .01 |  | |  |
| Condition×*5-HTTLPR* | | .99 | .80 | 4.00 | 680.00 | .53 | | .01 |  | |  |
| Condition×negative parenting | | .99 | .60 | 4.00 | 680.00 | .67 | | .00 |  | |  |
| Condition×positive parenting | | .99 | .85 | 4.00 | 680.00 | .50 | | .01 |  | |  |
| Condition×*5-HTTLPR*×negative parenting | | .98 | 1.21 | 6.00 | 680.00 | .30 | | .01 |  | |  |
| Condition×*5-HTTLPR*×positive parenting | | .99 | .54 | 6.00 | 680.00 | .78 | | .01 |  | |  |
| *Excluding children from non-European decent (n =360)* | | | | | | | | |  | |  |
| Condition | | .91 | 8.16 | 4.00 | 680.00 | .00 | | .05 | |  | |
| *5-HTTLPR* | | 1.00 | .11 | 2.00 | 340.00 | .90 | | .00 | |  | |
| Negative parenting | | 1.00 | .37 | 2.00 | 340.00 | .69 | | .00 | |  | |
| Positive parenting | | .98 | 4.42 | 2.00 | 340.00 | .01 | | .03 | |  | |
| Condition×*5-HTTLPR* | | .99 | .54 | 4.00 | 680.00 | .70 | | .00 | |  | |
| Condition×negative parenting | | 1.00 | .28 | 4.00 | 680.00 | .89 | | .00 | |  | |
| Condition×positive parenting | | .98 | 1.73 | 4.00 | 680.00 | .14 | | .01 | |  | |
| Condition×*5-HTTLPR*×negative parenting | | .97 | 1.49 | 6.00 | 680.00 | .18 | | .01 | |  | |
| Condition×*5-HTTLPR*× positive parenting | | .99 | .33 | 6.00 | 680.00 | .92 | | .00 | |  | |
| *Including a tri-allelic factor (n = 403 )* | | | | | |  | |  | |  | |
| Condition | | .93 | 6.23 | 4.00 | 662.00 | .00 | | .04 | |  | |
| Tri-allelic score | | .99 | 1.02 | 4.00 | 762.00 | .40 | | .01 | |  | |
| Negative parenting | | 1.00 | .78 | 2.00 | 331.00 | .46 | | .01 | |  | |
| Positive parenting | | .99 | 1.54 | 2.00 | 331.00 | .22 | | .01 | |  | |
| Condition×*5-HTTLPR* | | .98 | 1.04 | 8.00 | 662.00 | .41 | | .01 | |  | |
| Condition×negative parenting | | 1.00 | .43 | 4.00 | 662.00 | .79 | | .00 | |  | |
| Condition×positive parenting | | 1.00 | .26 | 4.00 | 662.00 | .90 | | .00 | |  | |
| Condition×*5-HTTLPR*×negative parenting | | .98 | .64 | 12.00 | 662.00 | .81 | | .01 | |  | |
| Condition×*5-HTTLPR*× positive parenting | | .99 | .41 | 12.00 | 662.00 | .96 | | .01 | |  | |
| *Including child age and gender (n = 405)* | | | | | |  | |  | |  | |
| Condition | | .98 | 1.57 | 4.00 | 768.00 | .18 | | .01 | |  | |
| *5-HTTLPR* | | 1.00 | .49 | 2.00 | 384.00 | .61 | | .00 | |  | |
|  | |  |  |  |  |  | |  | |  | |
| age | | 1.00 | .88 | 2.00 | 384.00 | .42 | | .01 | |  | |
| gender | | .99 | 1.32 | 2.00 | 384.00 | .27 | | .01 | |  | |
| Condition×*5-HTTLPR* | | .99 | .83 | 4.00 | 768.00 | .51 | | .00 | |  | |
| Condition×age | | .99 | .58 | 4.00 | 768.00 | .68 | | .00 | |  | |
| Condition×gender | | .96 | 4.10 | 4.00 | 768.000 | .00 | | .02 | |  | |
| Condition×*5-HTTLPR*×age | | .99 | .89 | 6.00 | 768.00 | .51 | | .01 | |  | |
|  | |  |  |  |  |  | |  | |
| Condition×*5-HTTLPR*×gender | | .88 | .42 | 6.00 | 768.00 | .87 | | .00 | |  | |
|  | | | | | | | | | | |  |

Table B. *Multivariate results auxiliary analyses affective reactivity to condition stimuli.*

| *Wilk’s Lambda* | | | | *F* | *df* | *dferror* | | *p* | | Partial η² |  |
| --- | --- | --- | --- | --- | --- | --- | --- | --- | --- | --- | --- |
| *Including one parent-child dyad per family (n = 404)* | | | | | | | | | |  |  |
| Condition | .96 | | | 4.45 | 4.00 | | 766.00 | | .00 | .02 |  |
| *5-HTTLPR* | 1.00 | | | .88 | 2.00 | | 383.00 | | .41 | .01 |  |
| Negative parenting | 1.00 | | | .10 | 2.00 | | 383.00 | | .90 | .00 |  |
| Positive parenting | .97 | | | 5.63 | 2.00 | | 383.00 | | .00 | .03 |  |
| Condition×*5-HTTLPR* | 1.00 | | | .31 | 4.00 | | 766.00 | | .87 | .00 |  |
| Condition×negative parenting | .98 | | | 1.62 | 4.00 | | 766.00 | | .17 | .01 |  |
| Condition×positive parenting | .99 | | | 1.05 | 4.00 | | 766.00 | | .38 | .01 |  |
| Condition×*5-HTTLPR*×negative parenting | .99 | | | .84 | 6.00 | | 766.00 | | .78 | .01 |  |
| Condition×*5-HTTLPR*×positive parenting | .99 | | | .89 | 6.00 | | 766.00 | | .50 | .01 |  |
| *Excluding children from non-European decent (n = 415)* | | | | | | | | | | |  |
| Condition | | | .95 | 5.05 | 4.00 | | 788.00 | | .00 | .03 |  |
| *5-HTTLPR* | | | 1.00 | .72 | 2.00 | | 394.00 | | .49 | .00 |  |
| Negative parenting | | | 1.00 | .02 | 2.00 | | 394.00 | | .98 | .00 |  |
| Positive parenting | | | .97 | 6.88 | 2.00 | | 394.00 | | .00 | .03 |  |
| Condition×*5-HTTLPR* | | | .99 | 1.17 | 4.00 | | 788.00 | | .32 | .01 |  |
| Condition×negative parenting | | | .98 | 1.74 | 4.00 | | 788.00 | | .14 | .01 |  |
| Condition×positive parenting | | | .99 | 1.53 | 4.00 | | 788.00 | | .19 | .01 |  |
| Condition×*5-HTTLPR*×negative parenting | | | .99 | .92 | 6.00 | | 788.00 | | .48 | .01 |  |
| Condition×*5-HTTLPR*× positive parenting | | | .99 | .74 | 6.00 | | 788.00 | | .62 | .01 |  |
| *Including a tri-allelic factor (n = 460)* | | | | | | | | | | |  |
| Condition | | | .96 | 4.89 | 4.00 | | 860.00 | | .00 | .02 |  |
| *5-HTTLPR* | | | 1.00 | .39 | 4.00 | | 860.00 | | .82 | .00 |  |
| Negative parenting | | | 1.00 | .50 | 2.00 | | 430.00 | | .61 | .00 |  |
| Positive parenting | | | .99 | 2.67 | 2.00 | | 430.00 | | .07 | .01 |  |
| Condition×*5-HTTLPR* | | | .99 | .47 | 8.00 | | 860.00 | | .88 | .00 |  |
| Condition×negative parenting | | | .99 | 1.07 | 4.00 | | 860.00 | | .37 | .01 |  |
| Condition×positive parenting | | | 1.00 | .32 | 4.00 | | 860.00 | | .86 | .00 |  |
| Condition×*5-HTTLPR*×negative parenting | | | .97 | 1.30 | 12.00 | | 860.00 | | .21 | .02 |  |
| Condition×*5-HTTLPR*× positive parenting  .94 | | .97 1.20 | | | 12.00 | | 860.00 | | .28 | .02 |  |
| *Including child age and gender (n = 460)* | | | | | | | | | | |  |
| Condition | | | .99 | 1.24 | 4.00 | | 878.00 | | .29 | 01 |  |
| *5-HTTLPR* | | | .99 | 1.76 | 2.00 | | 439.00 | | .17 | .01 |  |
| age | | | .99 | 2.20 | 2.00 | | 439.00 | | .11 | .01 |  |
| gender | | | .99 | 2.52 | 2.00 | | 439.00 | | .09 | .01 |  |
| Condition×*5-HTTLPR* | | | 1.0 | .12 | 2.00 | | 439.00 | | .89 | .00 |  |
| Condition×age | | | .98 | 1.82 | 4.00 | | 878.00 | | .12 | .01 |  |
| Condition×gender | | | .99 | 1.38 | 4.00 | | 878.00 | | .24 | .01 |  |
| Condition×*5-HTTLPR*×age | | | .98 | 1.22 | 6.00 | | 878.00 | | .30 | .01 |  |
| Condition×*5-HTTLPR*×gender | | | .99 | .85 | 8.00 | | 878.00 | | .56 | .01 |  |
